# A cross-country comparison of user experience of public autonomous transport

**DOI:** 10.1186/s12544-021-00477-3

**Published:** 2021-03-08

**Authors:** Mauro Bellone, Azat Ismailogullari, Tommi Kantala, Sami Mäkinen, Ralf-Martin Soe, Milla Åman Kyyrö

**Affiliations:** 1 Chalmers University of Technology, Hörsalsvägen 7a, 412 96 Göteborg, Sweden; 2 Metropolia University of Applied Sciences, Smart Mobility Innovation Hub, Leiritie 1, 01600 Vantaa, Finland; 3 FLOU Ltd., Pohjoisranta 14, 00170 Helsinki, Finland; 4 Tallinn University of Technology, Smart City Center of Excellence, Ehitajate tee 5, 19086 Tallinn, Estonia; 5 Metropolia University of Applied Sciences, RDI Services, Hämeentie 135D, 00560 Helsinki, Finland

**Keywords:** Autonomous vehicles, Electric minibuses, User experience

## Abstract

Autonomous solutions for transportation are emerging worldwide, and one of the sectors that will benefit the most from these solutions is the public transport by shifting toward the new paradigm of Mobility as a Service (MaaS). Densely populated areas cannot afford an increase in individual transportation due to space limitation, congestion, and pollution.

Working towards more effective and inclusive mobility in public areas, this paper compares user experiences of autonomous public transport across Baltic countries, with the final goal of gaining an increased insight into public needs. User experience was evaluated through questionnaires gathered along pilot projects implementing a public transportation line, using an automated electric minibus between 2018 and 2019. To have sufficient diversity in the data, the pilot projects were implemented in several cities in the Baltic Sea Area. The data analysed in this paper specifically refer to the cities of Helsinki (Finland), Tallinn (Estonia), Kongsberg (Norway), and Gdańsk (Poland).

Across all cities, passengers provided remarkably positive feedback regarding personal security and safety onboard. The overall feedback, which was very positive in general, showed statistically significant differences across the groups of cities (Kongsberg, Helsinki, Tallinn and Gdansk), partially explicable by the differences in the route design. In addition, across all cities and feedback topics, males gave a lower score compared to females. The overall rating suggests that there is a demand for future last-mile automated services that could be integrated with the MaaS concept, although demand changes according to socio-economic and location-based conditions across different countries.

**Supplementary Information:**

The online version contains supplementary material available at 10.1186/s12544-021-00477-3.

## Introduction

This paper analyses Mobility as a Service (MaaS) from the shared and automated urban transport perspective. As a concept, MaaS aims to revolutionise human mobility with a push towards subscription-based models. This change has effectively taken place in several fields, e.g. the media sector (music, news, books, and movies are increasingly consumed through subscription-based services as an alternative to the ownership model). The trillion-dollar-question is how this translates into the field of mobility, which is capital-intensive and subsidy-dominated. One of the potential milestones is last-mile urban transport that can integrate traditional transport service with novel technologies such as automated transport. In addition to all technical challenges and issues related to open-street automated mobility, a crucial aspect from the MaaS perspective is also to study user perception of security and safety onboard, after taking a ride.

The MaaS concept is largely based on shared mobility [2, 28], where automation can provide a significant contribution. One of the main advantages that autonomous vehicles bring to the urban environment is the use of shared autonomous vehicles (SAV). In the SAV paradigm, users are expected to share vehicles for on-demand transportation while optimizing the use of urban space, avoiding congestion, and minimizing risks. While new mobile technologies fostered the increase in use of shared vehicles services, such increase is still limited [16]. One of the reasons may be related to the actual accessibility of shared vehicles. In a recent study, Fagnant & Kockelman [10] simulate the effect of SAV in dynamic ride sharing. They conclude that the combined approach of SAV and dynamic ride sharing reduces waiting time and in-vehicle travel time respect to the current approach. Furthermore, in the study the authors suggest that private fleet operators of SAVs can be profitable. Dynamic ridesharing is, at least theoretically, an effective way to substitute manual driving, decrease risk of accidents, congestion and parking-related challenges in cities. The simulation assumes that most people are still able to go on their everyday personal trips (with rather minor changes in timing) by substituting personal cars with SAVs.

Although several predictions about the trend of autonomous driving have been done through the last decades, a large number of studies and stakeholders currently expect autonomous driving under restricted conditions running by 2020–2025, increasing the autonomy level to most of the driving conditions by 2025–2035. Full automation is only expected to reach 50% market share by 2050 (e.g. [14, 26]). The economic effects of full automation in transportation are analysed in Clements and Kockelman [6], concluding that the economic impact could reach $1.2 trillion per year in the US. This would involve cross-sector industries including automotive, electronics and software technology, trucking–freight movement, personal transport, auto repair, medical, insurance, legal profession, construction and infrastructure, land development, digital media, police and traffic violations, and oil and gas.

The Sohjoa Baltic project piloted last-mile integration of automated shuttle services opened up for all citizens as a free service in a close collaboration with local municipals. Shuttle passenger feedback was collected from late 2017 to late 2019 in four European cities (Kongsberg, Helsinki, Tallinn and Gdansk). Each pilot city carried a few thousand passengers and lasted for an average of 3–4 month with a weekly operational schedule of 20 h. This study compares the user feedback collected across the four cities, whereas pilot design differed slightly based on location. Out of over 15,000 people using the service, over 800 responded to the passenger feedback. Respondents covered various nationalities, gender, age and occupational groups. In general, the passenger feedback and personal feeling of safety and security on board is very positive, indicating demand for future last-mile automated services that could be integrated with the MaaS concept. On the other hand, socio-economic and location-based variations are to be expected.

The main research gap is that previous studies tend to focus on single-site pilot and/or similar automated pilots when analysing the passenger feedback, personal feeling of safety, and security on board. This paper, however, analyses passenger feedback with the same method across different pilot sites. The pilot sites were distributed across four geographic areas in 4 different cities in Finland, Poland, Norway and Estonia with different route specifications (e.g length, average speed, complexity of traffic etc), they were not homogeneous, and involved different AV shuttle buses (EasyMile and Navya). This setup, at least conceptually, should provide more accurate results, as it is possible to study safety and security on board independently from specific city, route specifications or shuttle bus.

The paper is organized as follows: Section 2 provides a review of the state of the art and other recent works in the same area. Section 3 describes our methodology for data gathering during the public surveys. Results from the surveys are thoroughly discussed in Section 4, including each individual city and a cross comparison among all the given sites.

## Related work

Although many studies on technological aspects of autonomous driving exist, only a few consider user experience. Only little data is available on public opinion, of which most is based on general surveys not linked to autonomous transportation pilot projects.

In 2014 an online survey was carried out in the biggest English-speaking countries, USA, UK and Australia, involving over 1500 respondents, finding that most of the people was already informed about autonomous vehicles and they had very high expectation about the technology [24].

An internet-based survey carried out in 2015 [14] came to the conclusion that fully automated transport could substitute manual driving during the next 3–4 decades, although manual driving was the most preferred mode. The survey involved 5000 participants from 100 countries. Interestingly, respondents were concerned about software hacking, legislative changes, and safety. Nordhoff et al. [17] presented the results of a large survey on 10,000 respondents via an on-line survey on random respondents in over 100 countries, the result is valuable and people was willing to take a ride in an autonomous bus, though they have not done yet. One important result was that the electric component was predominant in the choice of people, autonomous shuttles should be electric for most of respondents.

A conceptual model for user acceptance of automated driving vehicles was developed by Garidis et al. [11]. This model was tested through an online survey with 470 respondents in Germany, where respondents had no direct user experience. According to the study findings, safety had the most positive impact on the intention to use autonomous vehicles, followed by hedonistic reasons (fun to drive a self-driven vehicle). The most negative effect was associated with the desire of control. In Germany there were also other notable studies, Nordhoff et al. [18] present the result of a questionnaire on 384 people who had experienced an automated bus in Berlin. Their result also confirms that users are positive toward automated shuttles, although present some concern about speed and space limitation. A deeper survey on 30 respondents called to a 50-min interview [19], shown that there was an unrealistic expectation of the technological capabilities of automated buses, showing that the current technology is not yet mature.

Haboucha et al. [12] researched vehicle ownership preference based on 700 respondents living in Israel or North America. They developed a model for long-term choices and concluded that 44% of people would prefer to use regular vehicles. Potential users of automated transport were found to be young, educated, and spend more time travelling. Interestingly, Haboucha et al. [12] also observed that Israelis are more willing to switch to automated vehicles compared to individuals of North America.

Zmud et al. [29] applied a car-technology acceptance model and ran an online survey based on 500 residents in Austin, Texas. The study concluded that most people prefer individual self-driving vehicles to Car2Go or Uber solutions. Regarding travel innovation, the majority of people were conservative, most of the respondents would not change their place of residence, did not predict an increase in annual miles nor the number of vehicles owned. Bansal & Kockelman [3] conducted a more specific survey across Texas based on 1000 respondents, connecting willingness to pay with automation SAE levels 2–4. Keeping in mind that the study was published a few years back and therefore the absolute numbers might be inflated by now, they pointed out that people are willing to spend around $ 3000 extra for level 2 automation, $ 4500 for level 3 automation, and $ 7500 for level 4 automation on private vehicles.

Specific studies on public transport provide promising results. Dong et al. [9] performed a survey in Philadelphia (US) on roughly 900 university students and staff, concerning people’s willingness to switch to driverless buses. The survey concluded that over two-thirds of respondents were willing to use a driverless bus over a traditional one on condition that an operator would still be on board. In contrast, only 13% were willing to take the bus without any operator. This study is in line with Becker and Axhausen [4] suggesting a connection between the age of respondents and acceptance of autonomous transport.

Research on acceptance of autonomous buses by passengers was conducted from June 2015 to February 2016, within the CityMobil2 project involving driverless buses in the city of Trikala, Greece, [21]. The study provides important conclusions: residents were positive towards the use of automated buses considering them as part of the transition to future “smart cities”; according to the respondents, the transition has to be driven by the innovation content, and this may lead to a possible enhancement of public transport service and ticket price reduction; safety issues were discouraging people from using autonomous public services; part of the population was opposed to the automated transportation service due to ideological or political reasons connected to the fear of job losses.

A recent study on ride experience, conducted in Neuhausen am Rheinfall (Switzerland) on 957 passengers between November 2018 and January 2019, suggested that both comfort and safety were perceived very positive by respondents [27]. However, specific events causing discomfort were mentioned, including sudden braking and other critical situations. Such situations were mostly technical related issues, revealing a potential for development in the area. Salonen [22] analysed passenger experience of traffic safety and in-vehicle security after travelling on a driverless shuttle bus in Finland. Passenger safety was perceived as better, while personal security was perceived as weaker than in conventional buses. Kaye et al. [13] studied drivers’ intention toward highly automated vehicles (Level 4) and evaluated cross-country differences between respondents in Australia, France and Sweden. For this purpose, they performed an online survey with 1563 respondents, though most of respondents declared no prior experience on operating highly automated vehicles. Interestingly, individuals from France gave significantly greater intention to use highly automated cars compared to respondents from Australia and Sweden.

Although there are several studies that focus on the attitudes towards automated driving based on non-experimental surveys (e.g. [3, 9, 12–14]), there are rather limited perception studies that are based on the actual experiment of automated trials. According to Soe and Müür [25], one of the main concerns with the previously mentioned studies is that the survey respondents have not been introduced with existing technologies. Nevertheless, there are a few and relatively recent studies that have been based on the actual experience of AVs. For example, [15] developed a model regarding the acceptance of AVs and tested this in two European cities (La Rochelle in France and Lausanne in Switzerland). Only the respondents (349) that took the ride with automated vehicles on two pilot sites, were asked to fill in the survey. On the other hand, the explanatory power of the model applied was only 22% percent, meaning this model (most probably) did not capture all factors which influence an individual’s behavioural intentions to use AVs, e.g. it neglected on-board comfort and perceived feeling of safety. Salonen and Haavisto [23] interviewed people after taking a ride with AVs in Finland and concluded that people perceived AV-s mainly as safe and secure, although were concerned about AVs in traffic. The similar conclusion was with the study of Distler [8] - people felt secure on the automated bus, in general. There are also studies that focus on previous experience with AVs. For example, Pakusch [20] focused on the previous experience with fully autonomous transportation and Kaye et al. [13] were also mainly attracting the respondents with previous experience/ understanding of AVs.

It is worth mentioning that most the studies presented in this section were performed on a single line or single city. To the knowledge of the authors, to this date only little cross-country study on this topic exists, such studies mainly rely on internet-based surveys. This study therefore aims to contribute to the topic in this respect by addressing questions to passengers on board of autonomous shuttles in different cities of the Baltic sea area.

## Methodology

User experience and acceptance are the key requirements for wider utilization of autonomous public transport. During the pilots within the Sohjoa Baltic project, a survey was conducted to give insight regarding passenger experience on-board automated shuttles. The survey was designed together with the piloting cities Kongsberg, Helsinki, Tallinn and Gdansk during August–September 2018, with the external support from academic project partners (University of Gdańsk, Chalmers University and Metropolia), to review and refine the questions to serve each city’s interests and provide collected data to give a transnational perspective rather than national only. The questions were designed in English and then translated to local language from native speakers in each country to minimize differences in interpretation. The responsibility for the implementation of the survey data collection was a given to each pilot city safety operators. Regardless of the set goals for the number of answers, operating the AV safely was always their priority, and collecting data their secondary task. This may have also affected the number of responses to the questionnaire. Priority was given to make the survey quick and easy to fill in order to collect as many responses as possible. For this reason, the number of questions, and the depth of individual topics, had to be optimized.

The project Sohjoa Baltic (2017–2020) developed the knowledge and competencies required to deploy more sustainable and smarter automated last/first-mile service pilots for public transport needs in the cities of the Baltic Sea Region. Three cities were chosen for large-scale pilot projects: Kongsberg (Norway), Helsinki (Finland) and Tallinn (Estonia); and, more in small-scale, Gdansk (Poland) and Zemgale region (Latvia), while the sixth planned pilot in Vejle (Denmark) was cancelled due to national legislation issues. The Zemgale region pilot project was planned for spring 2020, but unfortunately, needed to be postponed, due to the Covid-19 pandemic.

All concluded pilot projects were conducted on open roads, in mixed traffic, and the ride was free of charge to all passengers. A safety driver onboard was ready to take control and drive manually when the used vehicle, a small (capacity up to 8 seats) automated (SAE-level 3/4) electric shuttle, was unable to perform in automated mode. Altogether over 15,000 passengers were carried on the pilot routes, each route was operated in cooperation with local transport authorities, and vehicles either from Navya or EasyMile. More information about the project and the pilot cities can be found in previous publications ([1], and [5]).

### Survey

The survey form comprised two parts. The first part was related to the bus ride and the general acceptance of robot buses, whereas the second was aimed to collect anonymous background information about the responder (gender, age group, occupation, level of education, etc.). Participants were able to skip any question according to their preference.

The survey was organized onboard during the ride, either online through provided tablets, with passengers own mobile devices, or offline through a paper form. Participating in the survey was purely voluntary and except for Kongsberg, no rewards were provided. Participants to the survey were the voluntary test-passengers on the robot bus, so sampling is non-random and results cannot be directly extended to the general public.

During the pilot project in Kongsberg, two separate surveys were organized. The first was organized by the Sohjoa Baltic project, the second by the local public transport authority. Surveys were conducted onboard the bus and at the bus stop. Unlike the other pilots, the participants in Kongsberg were able to opt into a chance to win an iWatch in a local lottery.

In Helsinki, the bus was equipped with two tablet computers to fill the online survey. The survey form was provided in Finnish and in English. It was also possible to translate the form using an online translator to other languages. The quality of the Finnish to English translation was found to be reliable and close to the original survey form, therefore differences in the responses in different languages are not expected.

The survey form in Gdansk was provided on paper and filled during or after the ride. The passengers were answering the survey individually, but the onboard safety driver was able to assist the passengers in case of problems.

The passengers in the Tallinn pilot were able to fill out the survey using their own mobile devices. Links to the survey were provided to participants along with a short leaflet about the pilot.

In the following the most relevant parts of the survey will be discussed, the full questionnaire is reported in Additional file 1.

#### Passenger safety

With autonomous driving still in its infancy, road safety is a topic followed closely by public, politics and researchers. When automated vehicles operate among others, and in normal traffic conditions, i.e. with other vehicles either autonomous or not, the probability of collisions and the impact of accidents is increased compared to operation in a closed environment. Due to differences in operating environments in between the pilot cities, variations in user experience is expected. The behaviour of a robotic vehicle can differ from a human driver, generating confusion and creating an uncomfortable or unsafe feeling about the ride, even if the accident rate does not increase or is even reduced. Passenger safety is understood here as the passengers’ subjective feeling of traffic safety on board an automated bus.

The robot buses used in this study are designed in such a way that any traffic risk, triggered by sensor input, automatically results in sudden braking. Thus, the passengers’ perception about safety can be altered by such hard braking, while also increasing the risk of falling for passengers standing in the bus or bumping into the interior parts of the bus.

Road safety experience was surveyed by asking each passenger to respond with a grade from 1 to 7 about the safety on board.

#### Personal security

Personal security on an autonomous vehicle is still a largely an unknown factor. In our study, it is defined as the passengers’ subjective feeling of security traveling with other passengers without the presence of a human driver, since the enclosed shared environment of an autonomous vehicle without a dedicated driver or supervisor might provide challenges to the personal security of the passengers. Experienced threats or perceived risks of safety both have a negative impact on the overall user experience and acceptance. Possible risks for personal security are, for instance, other passengers, people outside the vehicle, or cyber threats. The factors affecting the security were not surveyed.

All the pilot projects were organized with a safety operator on board, which may affect the perceived personal security. The topic was included in the survey nevertheless to provide a baseline for further pilots without safety operator on board, and to identify possible other issues related to security.

The personal security was evaluated by respondents on a scale from 1 to 7.

#### Importance of the safety operator

Due to safety, regulatory limits, and technical requirements, all pilot projects were conducted with the safety operator on board. For a wider use of autonomous public transport, the operator workload should be reduced, and at least a large part of the vehicles should be able to operate without any operator on board. The aforementioned factors road safety and personal security, as well as or various other factors of passenger user experience can be impacted by the presence of an operator in the vehicle.

The passengers’ readiness to use autonomous public transport without safety operator on board was assessed by respondents via four alternative options starting from “*No, never*” to “*Yes definitely*”.

#### Typical use cases

Automated minibuses used in the pilot projects differ from typical public transport vehicles and transit lines by numerous factors besides the autonomous driving, most importantly by their speed, route layout, capacity, and service areas. To investigate user perspective about possible use cases for the vehicles, and differences in priorities, survey participants were asked to list the most likely use cases they think the vehicles might have. The survey provided various options for potential use cases ranging from “*in bad weather*”, to “*in closed large areas*” (see question n. 4 in Additional file 1 for the full list of the options).

Additionally, the participants were able to provide own suggestions.

#### Suitability for vulnerable groups

Requirements especially for the safety-related factors increase for vulnerable groups, e.g. children. The participants were asked if the service would be suitable to be used by children going to/from school. Survey question n. 5 in additional file 1 is meant to aggregate various safety, security, and risk aversion topics to give an overview of how well the service is ranking on those topics. Moreover, the survey was conducted among voluntary participants of the pilot projects, thus the question gives some insight into how the participants think other people might react to these robotic vehicles.

#### Overall experience

The overall experience for passengers can vary greatly due to several factors that were not specifically surveyed. Therefore, passengers were asked to grade the overall experience in question n. 6 (see Additional file 1), on a scale from 1 to 5, where 1 was associated with “*very bad*”, and 5 was associated with “*very good*”.

#### Potential

The implementation of autonomous driving in stable and operational public transport is the goal of these pilot projects and technological development of autonomous vehicles in general. To assess the potential for such implementation, users estimated how frequently they would use such transportation for their daily commute (question n. 7 in Additional file 1). Clearly, the specific route may influence the answer to this particular question.

### Analysis method

The surveys were conducted in different languages and responses were translated to English for analysis. Some minor differences in the exact expression of the question and choices occurred. However, these minor differences are not expected to impact reliability of the data.

Gaussian normality cannot be assumed for any of the data. Thus, the non-parametric Kruskall-Wallis test was used for comparison of score distributions. Kruskall-Wallis test is a non-parametric rank-sum test extended to three groups. It allows comparison of three or more groups with non-normal and unequal size to be compared against each other. Kruskall-Wallis test discriminates the stochastic dominance in the data. In many cases Kruskall-Wallis test can be informally said to test the difference in medians of groups. It is a substitute for one-way ANOVA when assumptions for it cannot be satisfied [7].

Dunn’s test is a nonparametric pairwise multiple comparison, which can be used once a Kruskall-Wallis test has been rejected. As the Kruskall-Wallis test does not indicate which group(s) differ from others, Dunn’s test is used to run pairwise comparisons. Multiple pairwise comparison introduces a need to modify the interpretation of *p*-values, due to multiple comparisons changing the meaning of α - presenting the probability of rejecting the null hypothesis for one test. Different adjustment methods, such as Bonferroni, Holm, or Benjamini and Hochberg, can be used to modify the test to ensure correct interpretation. Holm correction is used in this study [7]..

In case of statistically significant differences were observed in our data, Dunn’s test was used for pairwise comparisons, and Dunn’s test for stochastic dominance with “Holm” p-value adjustment method for multiple comparisons. A significance level of 0.05 is used as threshold value for statistical significance. Choices were re-coded if necessary. Further, generalized linear regression was used to determine impact of background variables (age, occupation, education, gender, usage of public transport) on the overall experience score. In gender, empty answers were converted to “*Prefer not to say*” category. With other variables, empty values were converted to NA and omitted from analysis. For open questions, content analysis was used to cluster the wishes and comments of the passengers to main categories. All statistical analyses were done in R 3.6.2 in a 64-bit Windows 10 machine.

## Survey analysis and cross-country comparison

The number of collected responses varies by city, with 385 responded in Gdansk, 246 in Helsinki, 54 in Kongsberg, and 152 in Tallinn, for a total of 837 respondents. Not all responses are complete as respondents were not required to answer all questions, thus some passengers reply only to part of the questionnaire, though the missing rate was relatively low. Table 1 shows the percentage of missing data for each question, whereas Table 2 reveals that personal information has a higher missing data percentage. In all charts given here, the numbers are reported in percentage over the number of respondents to provide a fair comparison among the cities. The absolute number of respondents in densely populated cities is higher than, for instance, Kongsberg where the population is lower. However, an additional local transport authority survey in Kongsberg may have influenced the number of responses in this city (this survey got fewer responses). In Gdansk there was a paper-based feedback survey having high response rate.

**Table 1:** Percentage of missing data in demographic information

	Gdansk	Helsinki	Kongsberg	Tallinn	Total
Gender	0.00%	0.00%	0.00%	0.00%	0.00%
Age	2.08%	3.66%	0.00%	0.00%	2.03%
Education	3.38%	6.50%	0.00%	0.00%	3.46%
Occupation	3.90%	5.28%	11.11%	0.66%	4.18%
How often uses public transport	2.71%	4.88%	0.00%	0.00%	2.68%

**Table 2:** Percentage of missing data for each question in the questionnaire

	Gdansk	Helsinki	Kongsberg	Tallinn	Total
(Q1) Traffic safety	1.30%	0.81%	3.70%	0.66%	1.19%
(Q2) Personal security	0.78%	0.81%	0.00%	0.00%	0.60%
(Q3) Would use without operator	1.56%	0.81%	0.00%	0.00%	0.96%
(Q4) Use cases	0.52%	1.63%	0.00%	0.66%	0.84%
(Q5) Feasible for kids	0.52%	1.22%	0.00%	0.00%	0.60%
(Q6) Experience	1.56%	2.03%	0.00%	0.66%	1.43%
(Q7) How often as daily commute	1.04%	2.85%	0.00%	1.97%	1.67%

This survey does not aim to be a general indication about user experience, further research would be needed for the generalizability of this result. However, it surely provides an indication about the trend toward the increasing trust in autonomous mobility.

### Survey demographics

**Fig. 1:**
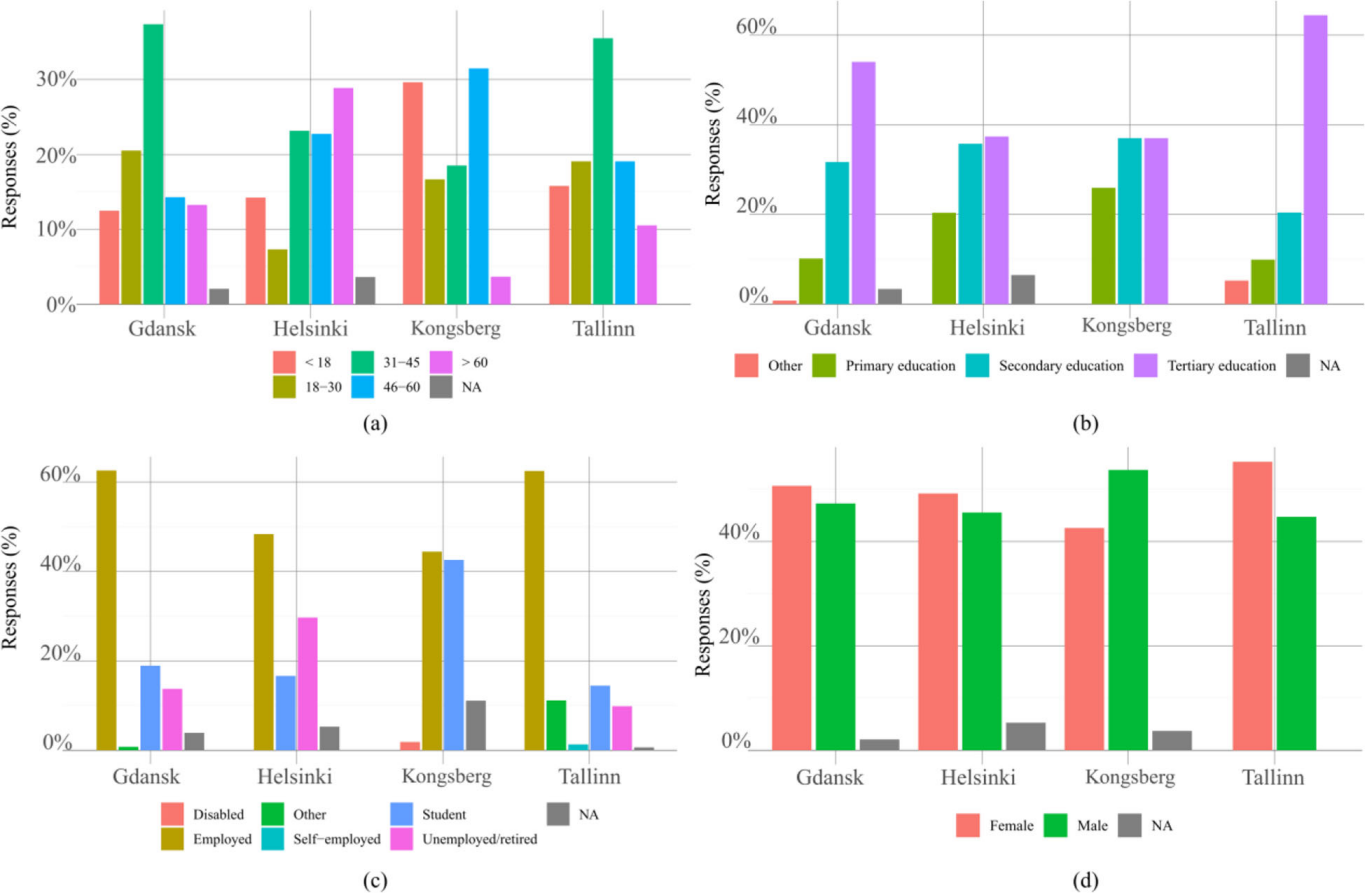
General information about the respondents: (**a**) age group, (**b**) education, (**c**) employment, and (**d**) gender

The distribution of respondents’ age groups across the different cities is shown in Fig. 1(a). The age groups are well distributed among the population, with some differences between cities. Further data can be seen in Table 3, reporting the exact valued for the distribution of age group and gender of respondents. The age distributions are roughly the same in Gdansk and Tallinn. In Helsinki, a higher proportion of respondents is over 60 years old. These differences are caused by the different areas where pilots were held. Aurinkolahti residential area in Helsinki has a high population of elderlies who can dedicate more time to fill the questionnaire. On the other hand, in Kongsberg the autonomous bus was used to connect the technology park to the central station, thus it was more used more frequently by a younger age group.

**Table 3:** Distribution of responses by age and gender per city

		Gdansk	Helsinki	Kongsberg	Tallinn	Total
Age	< 18	48	35	16	24	123
	18–30	79	18	9	29	135
	31–45	144	57	10	54	265
	46–60	55	56	17	29	157
	> 60	51	71	2	16	140
	Total	377	237	54	152	820
Gender	Female	195	121	23	84	423
	Male	182	112	29	68	391
	Not declared	8	13	2	0	23
	Total	385	246	54	152	837

In general, there was a high level of education among participants, see Fig. 1(b). Most respondents were employed. In Kongsberg, a high percentage of students results from the specific route chosen for the project.

Both genders were well represented in all cities, see Fig. 1(d). It is interesting to note that in all cities, except Kongsberg, the number of females is slightly higher. This reflects common statistics regarding of public transport usage per gender, which show that females typically use public transport more than males.

**Fig. 2:**
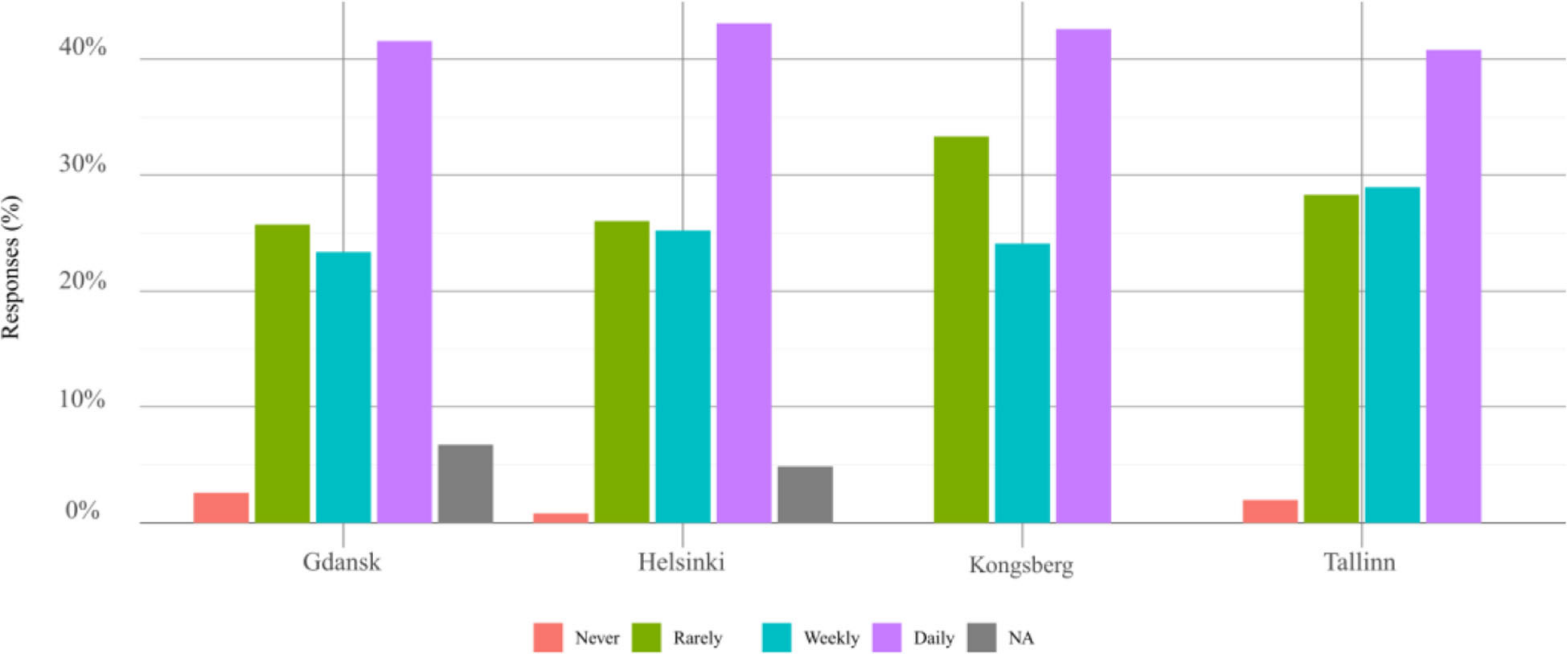
Overall frequency of public transport usage

The respondents declared that they use public transport rather often, on a daily basis or weekly, without relevant difference between the cities, as shown in Fig. 2. This information was collected to gain insight into the service quality users expect from automated public transport.

### Survey results

**Fig. 3:**
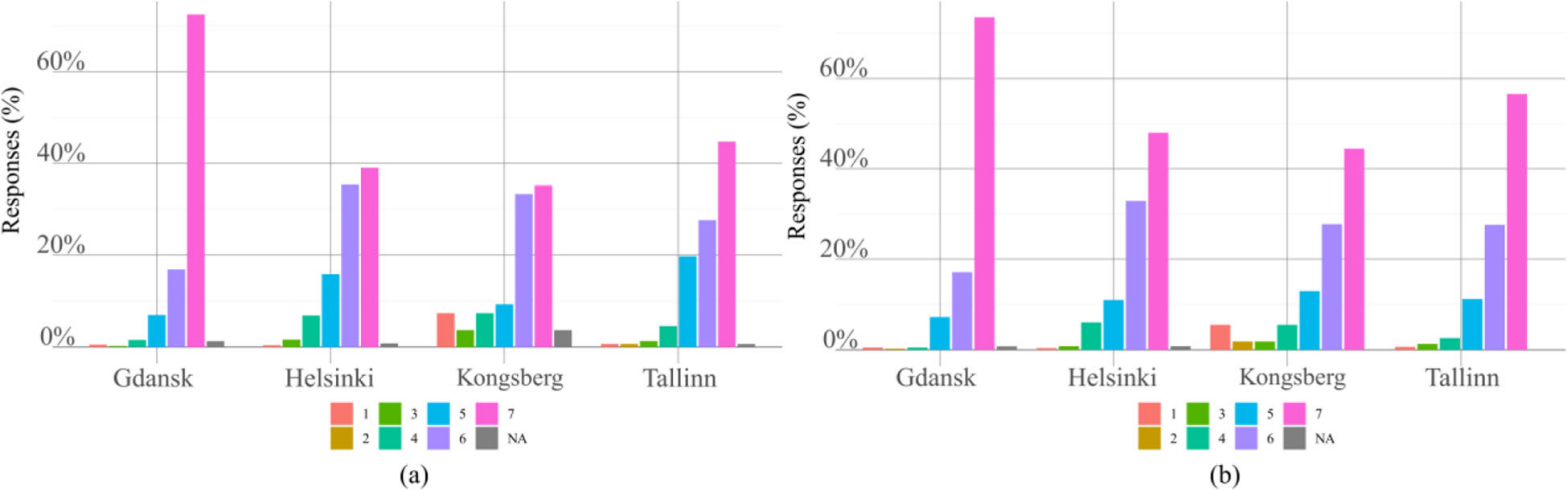
Distribution of scores per city for passenger safety in (**a**), and personal security in (**b**)

The answers of the questionnaire in Additional file 1 is here analysed to give an insight into user experience about public autonomous transportation. Perceived passenger safety scores (question n. 1 in Additional file 1) are shown in Fig. 3(a). The scores were remarkably high in all cities. The highest mean and median scores were given in Gdansk, with the difference in score distribution between Gdansk and other cities being statistically significant. Also, passenger safety received high scores in all cities with a median of 6, see Table 4. While the mean score in Kongsberg is lower than in Helsinki and Tallinn, the difference is not significantly different based on the tests. In Kongsberg, passenger safety received a number of extremely low scores, whereas in other cities extremely low scores are less frequent. The Kongsberg route design including a number of challenging factors, e.g. travel time and length were twice those of the other cities with more variable urban environments ranging from narrow street crossings to pedestrian paths.

**Table 4:** Mean, median and standard deviation values passenger safety and personal security scores given the score for each pilot city (scale from 1 -lowest to 7 -highest)

	City	Gdansk	Helsinki	Kongsberg	Tallinn
Passenger safety	Mean	6.60	6.02	5.62	6.06
	Median	7	6	6	6
	STD	0.82	1.04	1.72	1.11
Personal security	Mean	6.62	6.20	5.80	6.33
	Median	7	6	6	7
	STD	0.79	0.99	1.62	0.98

Personal security score, question n. 2 in Additional file 1 shown in Fig. 3(b), is comparable to the passenger safety score distribution, both are skewed heavily towards the positive end of choices. This is especially true in Gdansk, where score seven is very prominent, with the difference to other cities being statistically significant. Again, Kongsberg is the only city with a peak in the low scores of personal security.

**Fig. 4:**
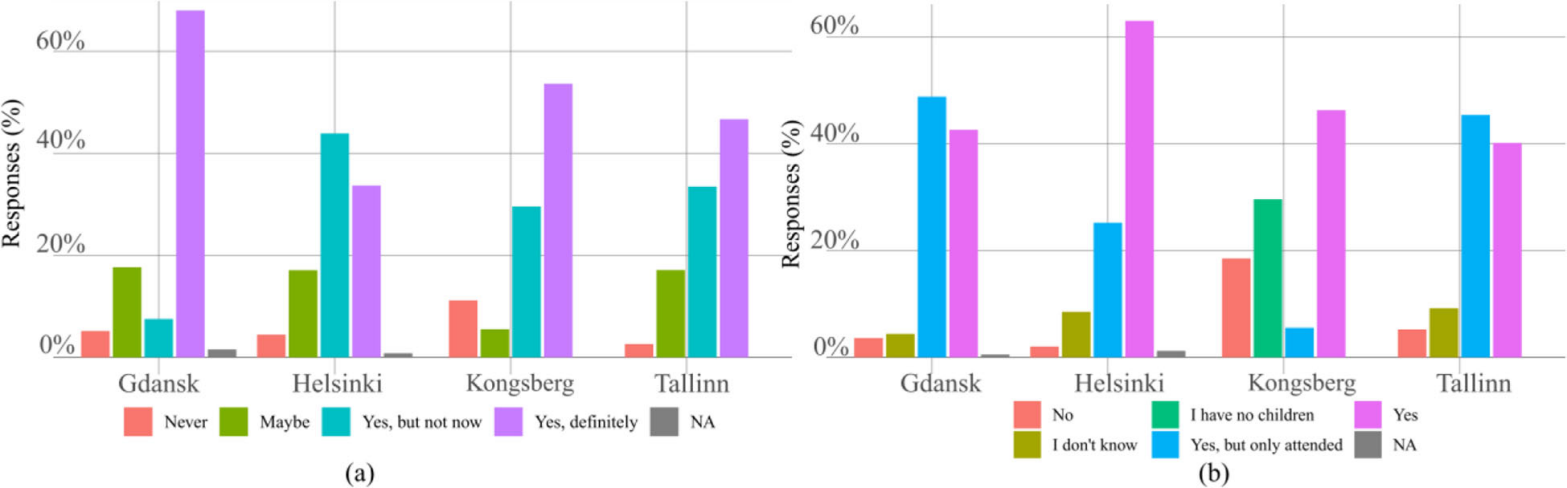
Responses to the questions: (**a**) use the service without operator, and (**b**) service suitable for children. Questions n. 3 and 5 respectively

Analysing the answers to question n. 3 in Additional file 1 shown in Fig. 4(a) appears that participants in Gdansk are much more likely to use the minibus without an operator. Kongsberg has the highest proportion of “*Never*” answers compared to others. Still, in all cities, the majority indicates willingness to use the automated minibus without an operator in the future. Differences between Gdansk and Tallinn and Gdansk and Helsinki are significant respectively.

The responses regarding children using the autonomous transportation by themselves, given in Fig. 4(b) and relative to question n. 5 in Additional file 1, differ among the pilot cities. For the analysis, the choice “*I have no children*” used only in Kongsberg was converted to “*I don’t know*” to create similar categories across all cities.

The distribution in Helsinki differs with statistical significance from the rest of the cities. Respondents in Helsinki are more open towards allowing children to use an autonomous bus by themselves. Responses in Kongsberg differ, although not significantly, from other cities, with a higher proportion of “*No*” answer. Also, in Kongsberg the proportion of “*Yes, but only attended*” is lower and the proportion of “*I don’t know*” higher than in other cities.

**Fig. 5:**
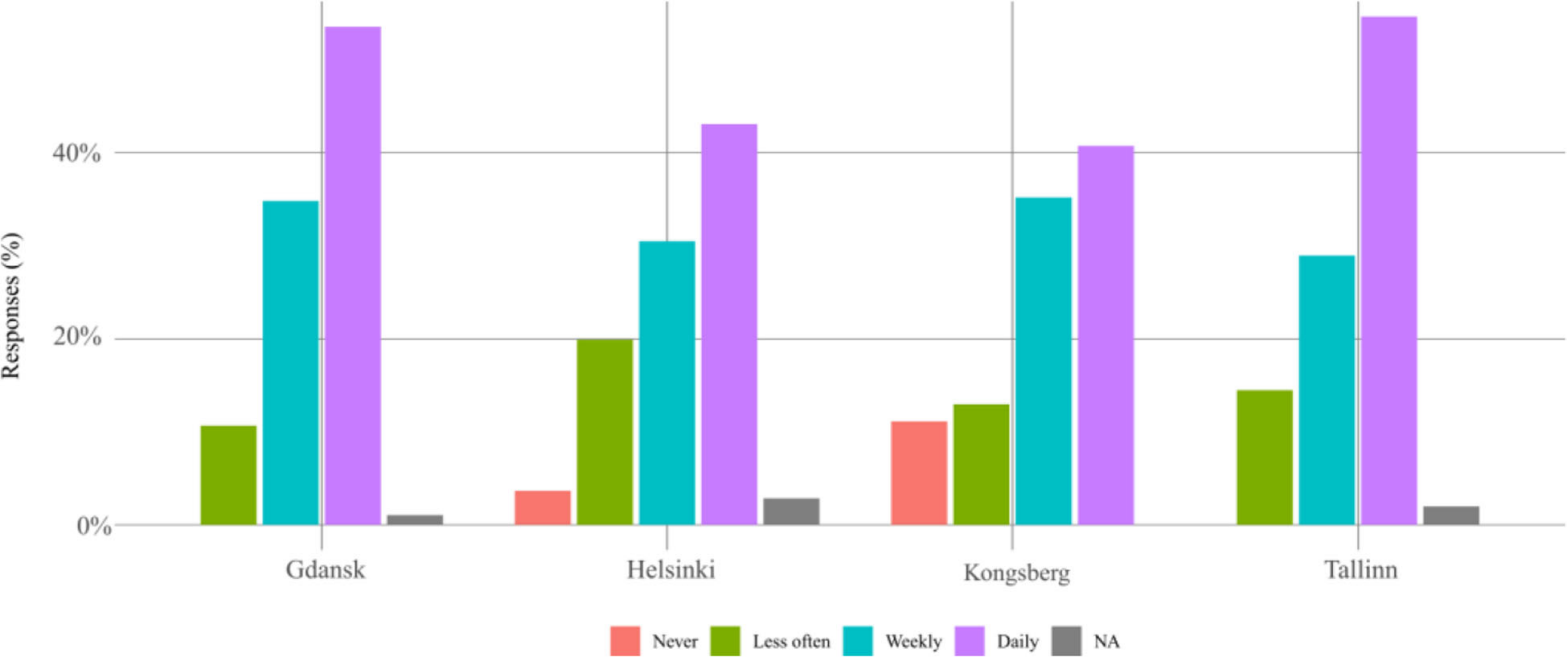
Distribution of answers to question n. 7 in Additional file 1, studying how often people would use the autonomous bus shuttle if it was available for daily commuting

The majority of respondents would use the autonomous bus for daily commuting if it was available, as shown in Fig. 5, this results from the answers to question n. 7. Interestingly, only in Helsinki and Kongsberg, some responded “*Never*”. In pairwise comparisons, Helsinki differs from Gdansk, and Tallinn, respectively; Kongsberg differs from Gdansk and Kongsberg-Tallinn pairwise comparison has *p*-value < 0.1. It seems that respondents in Helsinki and Kongsberg are slightly less open to use an autonomous vehicle for daily commuting.

No clear explanation for this preference was found from the pilot design. The minibuses used were not the same in these two cities (Navya - Helsinki and Tallinn; EasyMile - Gdansk and Kongsberg). However, the path length of the pilot was significantly longer in Kongsberg (5000 m) compared to 2000 m in the other cities, with an average speed of around 7–8 km/h across all sites. This also translated to travel time (with Kongsberg 37 min and other cities between 15 and 20 min on average). The main difference lies in the number of stops (Helsinki and Kongsberg had 7–8 stops; Tallinn and Gdansk 3–5). Most importantly, the pilot design differed between Gdansk/Tallinn and Helsinki/Kongsberg as well: in Gdansk and Tallinn, the pilot route was set in a low-intensity environment, whereas in Helsinki and Kongsberg the routes included more interaction with other traffic participants. For example, in Kongsberg the bus drove partially through pedestrian paths, and in Helsinki, the automated bus shared some of the stops with regular service lines.

No major differences in the potential use cases of autonomous minibuses between the cities were found. In Helsinki, the use for daily commute has a clearly lower proportion and in Kongsberg use in bad weather is slightly pronounced. Otherwise, all choices in all cities are nearly equally distributed. No statistically significant differences in the distribution of answers between the cities were found. Note that the question was a multiple-choice and most responses had multiple choices selected. Relatively few respondents chose “*Other*” or “*Never*” except in Kongsberg.

**Fig. 6:**
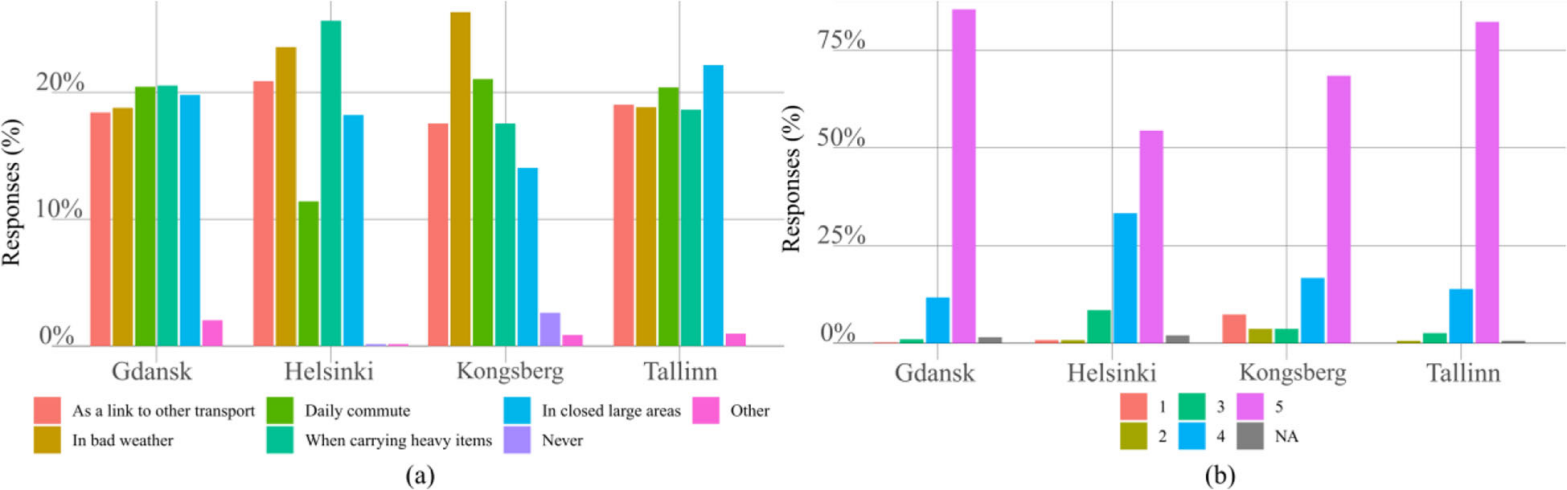
Distribution of relative scores for: (**a**) use cases, and (**b**) overall experience. Questions n. 4 and 6 respectively

Similarly, overall experience has been very positive, as indicated in Fig. 6(b) and Table 5 showing the mean, the median and the standard deviation of the score. Distributions in Gdansk and Tallinn differ with statistical significance from distributions in Helsinki to be more positive. However, in absolute terms, the differences are rather subtle and median values are equal in all cities.

**Table 5:** Mean and median of the distribution of the score about the overall experience

	City	Gdansk	Helsinki	Kongsberg	Tallinn
Overall experience	Mean	4.85	4.43	4.35	4.79
	Median	5	5	5	5
	STD	0.42	0.75	1.20	0.51

### Wishes from the respondents

Out of 837 responses to question n.8 in Additional files 1, 304 included a written wish or comment, which in the analysis were converted into 338 statements (summary shown in Table 6). Not all comments can be covered in this paper, however, the most important findings are presented next, especially ones with clear expressions of improvement ideas. One written comment can include several statements, such as “*increase speed*” and “*more space*”.

**Table 6:** Frequency of free comments per total responses for each city

City	Total responses	Comments	Frequency
Gdansk	385	177	0.46
Helsinki	246	37	0.15
Kongsberg	54	29	0.54
Tallinn	152	62	0.40
Total	837	304	0.36

Positive and negative statements without clear improvement ideas formed 31% of all statements, most of them being positive. Notably, 39% of comments in Gdansk, and 35% in Helsinki, did not include improvement ideas, compared to 17% in Tallinn and 20% in Kongsberg. Although the majority of the comments were positive, altogether 6 clearly negative comments were given. In Gdansk two (out of three) comments would have preferred extending the tram network. In Helsinki, the autonomous minibus was running late, and the participant could not test it. In Kongsberg, one respondent disliked the replacement of the driver. According to one respondent, only private sector should invest in the development. The amount of negative comments is nearly negligible overall.

The most frequent improvement idea related to the current low speed. A total of 42 comments (12%) included a wish for increased speed of the minibus. Interestingly, in Kongsberg the wish for increased speed formed 24% of all statements, whereas in Tallinn only 7% of statements desired higher speed.

Another clear group was formed by comments wishing for more routes. This category also includes wishes for specific routes in the city (such as city centre or airport), improving service level in remote areas and longer routes. This category forms 10% of all statements (*n* = 35).

People also wished for more buses in order to improve the frequency, and capacity and avoid waiting times, which was observed in 25 statements (7%). Interestingly, no one in Helsinki directly commented that more buses are needed (despite several queues of reaching half an hour during the peak time in July). Respondents also wished for more space inside the bus or more seats. Twenty-four such statements (7%) were identified across cities.

Five percent of the statements expressed hope for smoother operation. This might be mainly due to braking of the minibuses when obstacles are observed, especially in Kongsberg (17%) and Helsinki (12%). The passengers felt that the bus braked too hard. Due to this, some passengers also hoped for better passenger safety, mainly by confirming a need for seat belts during the ride (4% of statements).

Respondents also wished better interaction with traffic (4% of statements), which is partially related to the smoothness of the operation and better integration with other public transport modes (4% of statements), except in Kongsberg where no statements for interaction and integration were identified.

There were also statement categories wishing for autonomous minibuses replacing other modes of transport (3%), more independence (2%), more technological development in general (2%), on demand services (2%) and autonomous minibuses to be available as soon as possible (1%). Furthermore, 5% of the statements did not form a group, ranging from suggestions for using hydrogen as fuel, to having services on board.

## Conclusion and implications for future research

Overall, transportation in autonomous vehicles was perceived positively in the presented study. Passengers across all cities felt secure and safe on-board. The overall experience score is statistically significantly higher in Tallinn and Gdansk compared to Kongsberg and Helsinki. Interestingly, this result is not influenced by the choice of vehicle, as Kongsberg and Gdansk used an EasyMile bus, whereas Tallinn and Helsinki used a Navya shuttle bus. Similarly, this difference is not explained by city type (Tallinn and Helsinki are capital cities; whereas Kongsberg and Gdansk regional centres). The difference can be partially explained by the specific setup and route design. For example, Kongsberg and Helsinki routes included more interaction with other traffic participants, e.g. the bus drove partially through pedestrian paths in the city of Kongsberg, and shared bus stops with regular bus service in the city of Helsinki. In addition, on Helsinki and Kongsberg routes, the bus stopped approximately twice as often than in Tallinn and Gdansk. Kongsberg also stood out for twice the length of the route compared to other cities.

Furthermore, though not a focus of this paper, there may be socio-economic explanations: Tallinn and Gdansk represent post-soviet countries with a fast pace of transition over the past three decades, whereas Helsinki and Kongsberg come from a long period of socio-economic stability. An interesting follow up study can be perceived whether and how this affects user experience and acceptance.

Across all sites, males provide statistically significant lower overall experience scores compared to females. This might be explained by the higher expectations of males (e.g. higher speed), as males are more willing to take risks.

The information content in this study is to be considered as an indication of the trend of the public opinion and users’ attitude regarding autonomous mobility. However, the sample size and the specific responses limit the general validity of the study, more research would be needed in order to better frame the complicated topic of user experience, that, by definition, is based on subjects’ opinion about AVs. Finally, the information was acquired on pilot routes, the user experience in a real application of an integrated autonomous transportation system may differ from this result.

For the future research, the feedback survey results could be validated with more accurate sampling tools in order to generalise the results to the population level. In the current setting, surveys have bias towards the people interested in the AVs. Secondly, also the comparison could be done between the data received from the operators with the feedback surveys (e.g. how does human, technological or environmental etc. factors influence the feedback). Thirdly, a qualitative focus-group study could be performed to get in-depth insights from various socio-economic groups (such as elderly, people with disabilities, low-income earners etc).

## Supplementary Information

**Additional file 1.** Anonymous passenger survey.

## Data Availability

Data and material can be made available for reviewers upon request. All data is anonymized according to the data protection laws.
