# Supplementary material for: A cross-country comparison of user experience of public autonomous transport
Source: Eur Transp Res Rev. 2021 Mar 8;13(1):19. doi: 10.1186/s12544-021-00477-3 (PMC7938373; doi:10.1186/s12544-021-00477-3)
Supplement: Supplementary file 1 — Additional file 1. Anonymous passenger survey. [file 12544_2021_477_MOESM1_ESM.docx]

# Appendix A: Anonymous passenger survey

The Sohjoa Baltic project is conducting a research on automated bus user acceptance and experience. The research is confidential and collected data will only be used for statistical analysis. More about the Sohjoa Baltic, Interreg Baltic Sea Region funded project from http://sohjoabaltic.eu

1. How do you feel about traffic safety on-board? Please mark on a scale of 1 to 7.

Very unsafe 1 2 3 4 5 6 7 Very safe

1. How do you feel about your personal security on-board? Please mark on a scale of 1 to 7.

Very unsafe 1 2 3 4 5 6 7 Very safe

1. Would you also use the service with no operator on-board?
   1. Yes, definitely
   2. Yes, but not now
   3. Maybe
   4. No, never
2. When would you use this service? (More than one answer is allowed)
   1. in bad weather
   2. when carrying heavy items
   3. daily commute
   4. as a link to transport hubs/ other Public Transport options
   5. in closed large areas (e.g. campuses, industrial parks, airports, hospitals...)
   6. Never
3. Would it be feasible for children to use this vehicle to travel to/from the school?
   1. Yes
   2. Yes, but only attended
   3. No
   4. Don’t know
4. How would you describe your experience?

Very bad 1 2 3 4 5 Very good

1. If this service had been available as part of your daily commute, how often would you use it?
   1. Daily
   2. Weekly
   3. Less often
   4. Never
2. What wishes do you have about the future development on autonomous minibuses? Other feedback is also welcome!
